# Near-Infrared Transillumination Back Scattering Sounding—New Method to Assess Brain Microcirculation in Patients with Chronic Carotid Artery Stenosis

**DOI:** 10.1371/journal.pone.0061936

**Published:** 2013-04-17

**Authors:** Andrzej F. Frydrychowski, Pawel J. Winklewski, Arkadiusz Szarmach, Grzegorz Halena, Tomasz Bandurski

**Affiliations:** 1 Institute of Human Physiology, Medical University of Gdansk, Gdansk, Poland; 2 Department of Radiology, Medical University of Gdansk, Gdansk, Poland; 3 Department of Cardiovascular Surgery, Medical University of Gdansk, Gdansk, Poland; 4 Department of Nuclear Medicine and Radiological Informatics, Medical University of Gdansk, Gdansk, Poland; University of Cambridge, United Kingdom

## Abstract

**Purpose:**

The purpose of the study was to assess the responses of pial artery pulsation (cc-TQ) and subarachnoid width (sas-TQ) to acetazolamide challenge in patients with chronic carotid artery stenosis and relate these responses to changes in peak systolic velocity (PSV), cerebral blood flow (CBF), cerebral blood volume (CBV), mean transit time (MTT) and time to peak response (TTP).

**Methods:**

Fifteen patients with carotid artery stenosis ≥90% on the ipsilateral side and <50% on the contralateral side were enrolled into the study. PSV was assessed using colour-coded duplex sonography, CBF, CBV, MTT and TTP with perfusion computed tomography, cc-TQ and sas-TQ with near-infrared transillumination/backscattering sounding (NIR-T/BSS).

**Results:**

Based on the ipsilateral/contralateral cc-TQ ratio after acetazolamide challenge two groups of patients were distinguished: the first group with a ratio ≥1 and the second with a ratio <1. In the second group increases in CBF and CBV after the acetazolamide test were significantly higher in both hemispheres (ipsilateral: +33.0%±8.1% vs. +15.3%±4.4% and +26.3%±6.6% vs. +14.3%±5.1%; contralateral: +26.8%±7.0% vs. +17.6%±5.6% and +20.0%±7.3% vs. +10.0%±3.7%, respectively), cc-TQ was significantly higher only on the ipsilateral side (+37.3%±9.3% vs. +26.6%±8.6%) and the decrease in sas-TQ was less pronounced on the ipsilateral side (−0.7%±1.5% vs. −10.2%±1.5%), in comparison with the first group. The changes in sas-TQ following the acetazolamide test were consistent with the changes in TTP.

**Conclusions:**

The ipsilateral/contralateral cc-TQ ratio following acetazolamide challenge may be used to distinguish patient groups characterized by different haemodynamic parameters. Further research on a larger group of patients is warranted.

## Introduction

Occlusive cerebrovascular disease is associated with microvascular haemodynamic impairment. Chronic reductions in cerebral perfusion pressure are compensated by an increased oxygen extraction fraction and dilation of resistant arterioles [1], [2]. Pial window studies during acute reductions of perfusion pressure have revealed the dilation of arteries and veins on the surface of the brain in experimental animals [3], [4], [5]. However, acute brain ischaemia is associated with the up-regulation of endothelin type B (ET_B_) and 5-hydroxotryptamine type 1B (5-HT_1B_) receptors and/or reduced blood flow in rats, and related large cerebral artery vasoconstriction [6]. It is not clear whether the pial artery is affected or not. Nevertheless, if these findings are confirmed in humans, this means that during ischaemia arterial cerebral vessels are affected by several factors exerting contradictory effects. In humans the autoregulatory response to decreased cerebral perfusion pressure is estimated from indirect parameters such as cerebral blood flow (CBF), peak systolic velocity (PSV), cerebral blood volume (CBV), mean transit time (MTT), and time to peak response (TTP), to name a few. The main disadvantage of such an approach is that the abovementioned variables do not allow for the direct assessment of the status of small arterial cerebral vessels.

In the last decade, a new method based on infrared radiation (IR) called near-infrared transillumination/backscattering sounding (NIR-T/BSS) has been developed. NIR-T/BSS enables the assessment of changes in pial artery pulsation (cc-TQ), which reflect changes in pial artery compliance [7], [8], [9], [10]. In addition, NIR-T/BSS allows for measurement of the SAS width (sas-TQ), which reflects changes in CSF volume and/or intracranial pressure [11], [12], [13]. Contrary to near-infrared spectroscopy (NIRS), which relies on the absorption of infrared light (IR) by haemoglobin [14], NIR-T/BSS uses the SAS filled with translucent CSF as a propagation duct for IR [15], [16], [17], [18]. The main advantage of the NIR-T/BSS technology is that cc-TQ reflects the functional status of pial arteries, and thus allows for direct assessment of the generalized effect of various factors affecting pial arteries (i.e. decreased flow and/or oxygen supply, receptor up-regulation, etc.).

The acetazolamide test is often used to estimate autoregulatory reserve in patients with carotid artery stenosis. Cerebral perfusion of the brain tissue is measured before and after a vasodilatory challenge. Bokkers et al. [19] reported that there were no significant increases in vessel diameter in the hemisphere ipsilateral to the symptomatic ICA stenosis, whereas vessel diameter increased significantly in the proximal vasculature of the hemisphere contralateral to the ICA stenosis. Furthermore, the effect of acetazolamide on the side ipsilateral to the stenosis seems to be delayed [20]. As there might be differences in the response on the ipsilateral and contralateral sides to the stenosis, we hypothesized that the cc-TQ ipsilateral/contralateral ratio may help find patient subpopulations characterized by distinct cerebrovascular reactivity. The aim of this study was to assess cc-TQ and sas-TQ responses and compare them with established parameters such as PSV measured using colour-coded duplex sonography in the carotid artery and CBF, CBV, MTT and TTP measured via perfusion computed tomography (pCT) before and during an acetazolamide test in patients with chronic carotid artery stenosis.

## Methods

### Patients

Fifteen patients with carotid artery stenosis ≥90% on the ipsilateral side and <50% on the contralateral side were enrolled into the study. Diagnosis was based on ultrasonography examination and was confirmed using subtractive angiography. The patients' characteristics are presented in Table 1. In all patients, the duration of carotid artery stenosis was longer than 5 years. The experimental protocol and the study were approved by the ethical committee of the Medical University of Gdansk. All volunteers gave written informed consent to participate in the study. Patients with renal insufficiency were excluded.

**Table 1:** Patients' characteristics.

Patient No.	Age	Gender	Cerebrovascular History	Vascular Pathology	Concomitant Diseases	cc-TQ Ratio Ipsilateral/Contralateral
1.	70	Male	Previous left CAS	LICA restenosis >90%	Coronary artery disease, previous myocardial infarction, CABG and PTCA, hypertension, renal impairment	Baseline: 0.5 Diamox: 0.5
2.	69	Male	Recent stroke of the right hemisphere with transient contralateral hemiplegia	RICA stenosis >95%	Hypertension	Baseline: 1.3 Diamox: 1.1
3.	80	Female	Previous RICA surgical CEA	RICA restenosis >90%	Hypertension	Baseline: 0.8 Diamox: 0.7
4.	60	Female	Recent stroke of the left hemisphere with transient bracho-facial hemiparesis	LICA occlusion in intraoperative angiography	Hypertension, diabetes mellitus, coronary artery disease	Baseline: 0.9 Diamox: 1.2
5.	60	Female	Previous stent implantation for 99% stenosis in LICA	RICA stenosis >90%	Hypertension, coronary artery disease, multiple myocardial infarctions in medical history	Baseline: 0.5 Diamox: 0.6
6.	58	Male	Previous RICA surgical CEA	RICA restenosis >90%	Hypertension, dyslipidaemia, previous myocardial infarction, CABG and PTCA	Baseline: 1.0 Diamox: 1.0
7.	82	Male	Previous left subclavian artery revascularization with stent implantation	LICA stenosis >90%	Hypertension, coronary artery disease, peripheral arterial disease, previous PTCA	Baseline: 1.0 Diamox: 1.1
8.	70	Male	Previous TIA	LICA stenosis >90%	Hypertension, diabetes mellitus, coronary artery disease, previous myocardial infarction, PTCA, and lower limb endovascular and open revascularization	Baseline: 1.3 Diamox: 1.1
9.	70	Female	Previous TIA	LICA occlusion in intraoperative angiography	Hypertension, coronary artery disease, previous myocardial infarction	Baseline:1.3 Diamox: 1.2
10.	71	Male	Previous LICA surgical CEA with subsequent carotid artery stenting	RICA stenosis >95%	Hypertension, peripheral arterial disease, coronary artery disease, previous myocardial infarction	Baseline:1.6 Diamox: 1.3
11.	71	Male	Previous RICA surgical CEA	LICA stenosis >95%	Hypertension, coronary artery disease, previous myocardial infarction, CABG and PTCA, previous peripheral vascular surgery	Baseline: 0.9 Diamox: 1.4
12.	66	Male	Ischaemic stroke of the left hemisphere with contralateral hemiparesis and aphasia. Previous surgical LICA CEA	LICA restenosis >95%	Hypertension	Baseline:1.6 Diamox: 1.3
13.	81	Male	Previous RICA surgical CEA	LICA stenosis >90%	Hypertension, dyslipidaemia, coronary artery disease. Previous PTCA	Baseline: 0.7 Diamox: 0.7
14.	68	Male	Ischaemic stroke of the right hemisphere	RICA stenosis >90%	Hypertension	Baseline:0.8 Diamox: 1.0
15.	71	Female	Three strokes in the past with aphasia and transient right-sided plegia	LICA stenosis >90%	Hypertension, diabetes mellitus, atrial fibrillation	Baseline: 1.1 Diamox: 1.2

cc-TQ–pial artery pulsation (cardiac component of the transillumination quotient); CABG–coronary artery bypass grafting; CAS–carotid artery stenting; CEA–carotid endarterectectomy; LICA–left internal carotid artery; PTCA–percutaneous transluminal coronary angioplasty; RICA–right internal carotid artery; TIA–transient ischaemic attack.

### Doppler examination

Extracranial vascular pathology was assessed via continuous-wave Doppler and colour-coded duplex sonography of the carotid artery (5–13 MHz linear-array transducer, ALPHA 6, ALOKA, Hitachi, Zug, Switzerland). The degree of carotid artery stenosis was quantified according to North American Symptomatic Carotid Endarterectomy Trial (NASCET) criteria. We assessed the PSV of the internal carotid artery (ICA). The Doppler examinations were performed while the patients were in the supine position. Each patient underwent a resting flow study, and 20 minutes after the acetazolamide test (injection of 1.0 g Diamox i.v.).

### Computed tomography

Cerebral perfusion computed tomography (pCT) was performed on a CT unit equipped with a 64-detector row scanner (LightSpeed, GE Medical Systems, Milwaukee, Wisconsin, US). After unenhanced CT of whole brain eight adjacent 5 mm thick sections were selected starting at the level of the basal ganglion. A bolus of 40 mL of nonionic iodinated contrast medium (OPTIRAY 350; Mallinckrodt, Quebec, Canada) was injected at a rate of 4 mL/s into an antecubital vein using a power injector and with additional pushing using 40 mL of saline at a rate of 4 mL/s. At 5 seconds after initiation of the injection, a cine (continuous) scan was initiated with the following parameters: 80 kVp, 180–200 mAs, 8×5 mm thick sections, time resolution 0.5 s, total images 712, matrix: 512×512 pixels, acquisition time 45 seconds. CT perfusion data were analysed on an imaging workstation (Advantage Windows 4.4; GE Medical Systems, Milwaukee, Wisconsin, US) with perfusion analysis software (Perfusion 4, GE Medical Systems, Milwaukee, Wisconsin, US). We calculated the CBF, CBV, MTT and TTP and displayed each parameter as an 8-slice colour map. Regions of Interest (ROIs) were located in the frontal lobe cortex of each hemisphere to collect information from the area analysed by NIR-T/BSS sensors. Leptomenigial anastomoses were not assessed. Each patient underwent a computed tomography study before and 20 minutes after an acetazolamide test (injection of 1.0 g DIAMOX i.v.). All calculations were performed by the same experienced radiologist (AS). To minimize bias or systemic error the responsible radiologist was requested to assess patients in a blinded way, including radiological assessment of several patients suffering from other pathologies with normal CBF and CBV.

### Angiography

Confirmatory angiography was performed prior to carotid artery stenting. Patients were considered for this study when the internal carotid artery stenosis exceeded 90%, and the stenosis on the contralateral side was less than 50%. Patients who did not fulfil these criteria were excluded from this study. All procedures were performed by an experienced endovascular surgeon. Pre-procedurally all patients were on aspirin, and received clopidogrel (75 mg) at least 3 days before the procedure. All procedures were performed under local anaethesia, and no sedation was used. In all cases transfemoral access was chosen. Under local anaethesia a 6F sheath was inserted into the femoral artery and 5000 units of unfractionated heparin were given. A pigtail catheter was positioned in the ascending aorta and arch angiography was performed, revealing arch anatomy and its branches. Selective angiography of the target vessel was then performed to reveal the size of the carotid lesion, degree of stenosis, morphology of the internal carotid artery and its contribution to the circle of Willis.

### NIR-T/BSS

Changes in the amplitude of pial artery pulsation and in the width of SAS with NIR-T/BSS were recorded using a head-mounted SAS–100 NIR-T/BSS device (NIRT sp. z o.o., Wierzbice, Poland). The sensor unit consists of the emitter (E) and two photo-sensors located at different distances from the emitter. The NIR-T/BSS emitter is a near-infrared light-emitting diode (LED). The proximal sensor (PS) is located close to the emitter, while the distal sensor (DS) is located further away from the emitter. The stream of IR generated by the emitter penetrates the highly perfuse layer of the skin of the head, the skull bones and the SAS. The stream of radiation reflects from the surface of the brain and reaches the sensors, crossing the aforementioned layers of tissues in reverse order. Signals from the sensors undergo analogue–digital conversion in a specialised data acquisition system and are recorded on the microcomputer's hard disk for subsequent analysis with on-line presentation on the computer monitor.

The theoretical and practical foundations of the NIR-T/BSS method were determined in model studies conducted by our team [15], [16], [17], [18]. Briefly, a signal received by the DS is divided by the signal received by the PS. Such division reduces proportional factors that affect each of the two signals in an identical way, because the quotient of these factors assumes the value 1. Both the dividend, i.e., the power of the DS signal, and the divisor, i.e., the power of the PS signal, are influenced by the width of the SAS, and also by any factor capable of changing that width. Therefore, the quotient of the two signals, called the **transillumination quotient (TQ)**, is sensitive to changes in the width of the SAS. The oscillations in TQ have their origin in different modulation of the PS and DS signals, namely in the modulation of the DS signal on its way through the SAS. This arises because only the DS receives radiation propagated within the SAS. Propagation of IR in the skin and bone is much worse than in the clear translucent cerebrospinal fluid (CSF) contained in the SAS, and with the DS placed far enough away from the emitter, no radiation propagated in the superficial tissue layers can reach the DS [15], [16], [17], [18]. The power of the IR stream reaching the DS is directly proportional to the width of the SAS. The wider the SAS, or the propagation duct, the more radiation reaches the DS and the greater the signal from that sensor, which is the dividend in the calculation of the TQ [15], [16], [17], [18]. Conversely, the power of the IR stream reaching the PS is inversely proportional to the width of the SAS. Thus, the wider the SAS, the longer the distance between the inner skull bone surface and the brain at which IR energy is dissipated, and therefore the less IR follows the return route to the PS. The narrower the SAS, the closer the reflecting surface of the brain is to the PS and the more IR reaches that sensor.

Thus, in the **transillumination quotient (TQ)** we can identify three main components:

1. A **constant** or a **non-pulsatile component**, further referred to as the **subarachnoid component (sas-TQ)**; its value depends on the width of the CSF-filled SAS;
2. **Slow-variable pulsation**, further referred to as the **subcardiac component (scc-TQ)**; including, but not limited to, pulsation of a respiratory origin;
3. **Fast-variable pulsation**, further referred to as the **cardiac component (cc-TQ)**; resulting from heart-generated arterial pulsation that is the cause of fast oscillations in the width of the SAS.

For further analysis, the first harmonic of the arterial pulsation-dependent oscillations of TQ was extracted through appropriate filtering, along with its modulation. Modulation of that harmonic is a fast-variable component (or cardiac component) of the principal, second, and third harmonics of the cardiac component waveform, respectively.

Heart rate (HR) was calculated from cc-TQ tracings.

### Statistical analysis

Shapiro-Wilk, Mann-Whitney U and ANOVA tests were used to analyse the differences between average values. Changes in cc-TQ, sas-TQ, MTT, CBV, CBF, TTP PSV and HR responses were compared to baseline values and between hemispheres. Correlation and regression analysis was performed to assess interdependences between cc-TQ, sas-TQ, MTT, CBV, CBF, TTP PSV and HR. Before and after the acetazolamide test subgroup analysis was performed to find the cc-TQ ipsilateral/contralateral ratio to best differentiate patient populations with respect to MTT, CBV, CBF, TTP and PSV values. All statistical calculations were performed using the Statistics for Windows 8.0 software.

## Results

**Figure 1:**
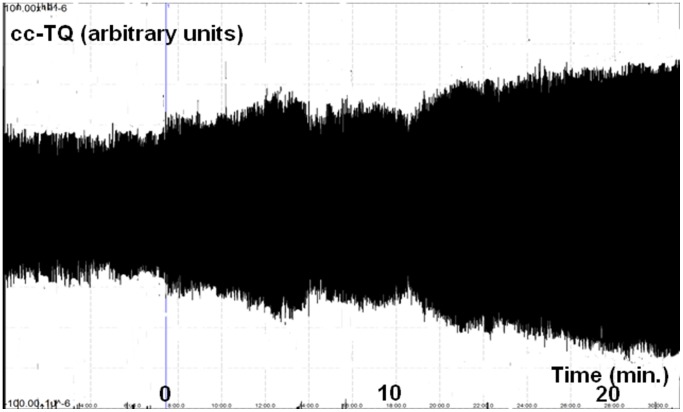
Representative cc-TQ recording before and after acetazolamide challenge. Vertical blue line indicates time of acetazolamide administration. cc-TQ–pial artery pulsation (cardiac component of the transillumination quotient).

The data collected from all 15 participants are summarized in Table 2. Typical cc-TQ tracing after acetazolamide administration is presented in Figure 1. Based on the cc-TQ following acetazolamide challenge we distinguished two groups of patients: the first group with an ipsilateral/contralateral cc-TQ ratio ≥1 and the second with a cc-TQ ratio <1. These two groups presented different characteristics at baseline and following the acetazolamide test. The first group consisted of 10 patients and the second of 5 patients. We found no direct interdependences between cc-TQ or sas-TQ and CBF, CBV, TTP or MTT.

**Table 2:** Mean (± SE) values of cc-TQ, sas-TQ, MTT, CBV, CBF, TTP and PSV before and after acetazolamide challenge in hemisphere ipsilateral and contralateral to stenosis. The whole patient population (n = 15).

Variable	Baseline		Acetazolamide challenge	
	Ipsilateral	Contralateral	Ipsilateral	Contralateral
**cc-TQ** (arbitrary units)	69.7±6.8	73.3±8.0	84.0±9.3^***^	94.6±13.6^***^
**sas-TQ** (arbitrary units)	10612.9±982.4	10241.3±1311.3	9865.9±998.8*	9346.1±1298.7^**^
**MTT** (s)	6.7±0.2	6.41±0.1	6.42±0.3^NS^	5.95±0.1^**^
**CBV** (ml/100 g)	2.07±0.1	2.0±0.1	2.42±0.2^***^	2.29±0.1^***^
**CBF** (ml/100 g/min.)	22.7±0.9	22.9±0.8	27.5±1.5^***^	27.8±1.2^***^
**TTP** (s)	26.4±1.0	26.0±1.1	24.7±1.0^***^	24.4±1.0^***^
**PSV** (cm/s)	126.2±37.6	79.2±8.0	167.3±33.9^**^	±8.0^***^
**HR** (beats/min.)	74.3±3.5		74.1±3.5^NS^	

* p<0.05, ^**^p<0.01, ^***^p<0.001, ^NS^not statistically significant, acetazolamide challenge versus baseline.

cc-TQ–pial artery pulsation (cardiac component of the transillumination quotient); sas-TQ–the subarachnoid width (subarachnoid component of the transillumination quotient); MTT–mean transit time; CBV–cerebral blood volume; TTP–time to peak; PSV–peak systolic velocity; HR–Heart Rate; SE–standard error; ml–millilitres; g–gram; min.–minutes; s–seconds; cm–centimetres.

### Ipsilateral/contralateral cc-TQ ratio ≥1

At baseline there were no differences in cc-TQ, sas-TQ, MTT, CBV or CBF between sides, while TTP and PSV were lower on the contralateral side. Acetazolamide evoked an increase in cc-TQ, CBV, CBF and PSV. TTP and sas-TQ diminished. The decrease in MTT was statistically significant only on the contralateral side. Following the acetazolamide test cc-TQ and PSV were higher on the ipsilateral side. The results of this group are summarized in Table 3.

**Table 3:** Mean (± SE) values of cc-TQ, sas-TQ, MTT, CBV, CBF, TTP and PSV before and after acetazolamide challenge in hemisphere ipsilateral and contralateral to stenosis. Patient population with cc-TQ ipsilateral/contralateral ratio ≥1 (n = 10).

Variable	Baseline		Acetazolamide challenge	
	Ipsilateral	Contralateral	Ipsilateral	Contralateral
**cc-TQ** (arbitrary units)	71.4±6.7	58.6±7.3	90.4±8.8^**^	74.3±7.0^## ***^
**sas-TQ** (arbitrary units)	10595.3±1083.9	10407.1±1433.4	9514.6±979.2^***^	9220.1±1358.4^**^
**MTT** (s)	6.9±0.3	6.5±0.2	6.7±0.4^NS^	6.0±0.2*
**CBV** (ml/100 g)	2.1±0.2	2.0±0.1	2.4±0.2*	2.2±0.1*
**CBF** (ml/100 g/min.)	22.9±1.3	22.7±0.8	26.4±2.1*	26.7±1.7*
**TTP** (s)	26.4±1.3	25.6±1.5$	24.5±1.2^***^	23.9±1.2^***^
**PSV** (cm/s)	131.8±56.2	80.0±7.6$	±50.2*	121.1±7.1# ***

$ p<0.05 baseline: ipsilateral vs contralateral;

# p<0.05, ^##^p<0.01 acetazolamide challenge: ipsilateral versus contralateral;

* p<0.05, ^**^p<0.01, ^***^p<0.001, ^NS^not statistically significant, acetazolamide challenge versus baseline.

cc-TQ–pial artery pulsation (cardiac component of the transillumination quotient); sas-TQ–the subarachnoid width (subarachnoid component of the transillumination quotient); MTT–mean transit time; CBV–cerebral blood volume; TTP–time to peak; PSV–peak systolic velocity; SE–standard error; ml–millilitres; g–gram; min.–minutes; s–seconds; cm–centimetres.

### Ipsilateral/contralateral cc-TQ ratio <1

At baseline there were no differences in sas-TQ, MTT, CBV, CBF, TTP or PSV between sides, while cc-TQ was significantly higher on the contralateral side. Acetazolamide evoked an increase in CBV, CBF and PSV. cc-TQ increased significantly only on the ipsilateral side. MTT, TTP and sas-TQ remained the same. There were no differences between the ipsilateral and contralateral side following the acetazolamide test. The results of this group are summarized in Table 4.

**Table 4:** Mean (± SE) values of cc-TQ, sas-TQ, MTT, CBV, CBF, TTP and PSV before and after acetazolamide challenge in hemisphere ipsilateral and contralateral to stenosis. Patient population with cc-TQ ipsilateral/contralateral ratio <1 (n = 5).

Variable	Baseline		Acetazolamide challenge	
	Ipsilateral	Contralateral	Ipsilateral	Contralateral
**cc-TQ** (arbitrary units)	66.4±12.0	102.8±7.3$	91.2±19.6*	135.2±33.2^NS^
**sas-TQ** (arbitrary units)	10648.0±2201.3	9909.6±2960.9	10568.6±2444.5^NS^	9598.2±3062.9^NS^
**MTT** (s)	6.22±0.2	6.3±0.2	5.9±0.3^NS^	5.8±0.3^NS^
**CBV** (ml/100 g)	1.9±0.1	2.0±0.2	2.4±0.1^**^	2.4±0.1*
**CBF** (ml/100 g/min.)	22.4±1.3	23.5±1.7	29.8±1.2^**^	29.8±0.8^**^
**TTP** (s)	26.5±1.8	26.7±1.8	25.0±1.7^NS^	25.5±1.6^NS^
**PSV** (cm/s)	115.0±26.3	±21.7	149.5±26.4*	127.5±22.9*

$ p<0.05 baseline: ipsilateral vs contralateral;

* p<0.05, ^**^p<0.01, ^NS^not statistically significant, acetazolamide challenge versus baseline.

cc-TQ–pial artery pulsation (cardiac component of the transillumination quotient); sas-TQ–the subarachnoid width (subarachnoid component of the transillumination quotient); MTT–mean transit time; CBV–cerebral blood volume; TTP–time to peak; PSV–peak systolic velocity; SE–standard error; ml–millilitres; g–gram; min.–minutes; s–seconds; cm–centimetres.

### cc-TQ ratio ≥1 versus cc-TQ ratio <1

In the group of patients with an ipsilateral/contralateral cc-TQ ratio <1, the increases in CBF and CBV following the acetazolamide test were significantly higher in both hemispheres, cc-TQ was significantly higher only on the ipsilateral side and the decrease in sas-TQ was less pronounced on the ipsilateral side. A detailed comparison of percentage changes in both groups is presented in Table 5.

**Table 5:** Comparison of mean (± SE) percentage changes after acetazolamide challenge versus baseline in patients with ipsilateral/contralateral cc-TQ ratio ≥1 and <1.

Variable	cc-TQ ≥1		cc-TQ <1	
	Ipsilateral	Contralateral	Ipsilateral	Contralateral
**cc-TQ** (arbitrary units)	+26.6%±8.6%	+26.8%±8.2%	+37.3%±9.3%*	+31.5%±6.0^NS^
**sas-TQ** (arbitrary units)	−10.2%±1.5%	−11.4%±2.7%	−0.7%±1.5%*	−3.1%±5.7%^NS^
**MTT** (s)	−2.9%±3.8%	−7.7%±2.1%	−5.1%±2.5%^NS^	−7.9%±3.5%^NS^
**CBV** (ml/100 g)	+14.3%±5.1%	+10.0%±3.7%	+26.3%±6.6%*	+20.0%±7.3%*
**CBF** (ml/100 g/min.)	+15.3%±4.4%	+17.6%±5.6%	+33.0%±8.1%*	+26.8%±7.0%*
**TTP** (s)	−7.2%±1.1%	−6.6%±0.8%	−5.7%±3.8%^NS^	−4.5%±2.4%^NS^
**PSV** (cm/s)	+33.8%±15.4%	+51.4±8.3%	+30.0%±11.8%^NS^	+64.5%±20.0%^NS^

* p<0.05, ^NS^not statistically significant, group of patients with ipsilateral/contralateral cc-TQ ratio ≥1 versus group of patients with cc-TQ <1.

cc-TQ–pial artery pulsation (cardiac component of the transillumination quotient); sas-TQ–the subarachnoid width (subarachnoid component of the transillumination quotient); MTT–mean transit time; CBV–cerebral blood volume; TTP–time to peak; PSV–peak systolic velocity; SE–standard error; ml–millilitres; g–gram; min.–minutes; s–seconds; cm–centimetres.

## Discussion

To the best of our knowledge this is the first study using NIR-T/BSS technology to assess changes in pial artery pulsation and the subarachnoid width in patients with cerebrovascular pathology. The main finding of this study is that, based on the cc-TQ ratio between the sides ipsilateral and contralateral to the stenosis, two groups of patients with different haemodynamic characteristics can be distinguished.

The investigated group of patients was heterogeneous in terms of medical history, including previous history of stroke and carotid artery endarterectomy (CEA) and stenting (CAS). The inclusion criteria were carotid stenosis ≥90% on the ipsilateral side and less than 50% on the contralateral side. All subjects suffered from concomitant diseases. The study population was representative for a group of patients with chronic carotid artery stenosis (Table 1). Quite surprisingly the population appeared very homogeneous with respect to low CBV and CBF values and as a consequence a relatively high CBF/CBV ratio (Table 2). Patients with low CBF and CBV values have already been described in the literature, but usually did not exceed 20–30% of the total investigated population [21], [22], [23], [24], [25]. To minimize bias or systemic error the responsible radiologist was requested to assess patients in a blinded way, including the radiological assessment of several patients suffering from other pathologies with normal CBF and CBV (data not shown).

A high CBF/CBV ratio due to low CBV suggests that patients were relatively well adapted to low CBF [22]. The lack of significant differences at baseline in terms of MTT, CBF, CBV and sas-TQ between the ipsilateral and contralateral hemisphere may confirm this hypothesis. Deydren et al. [2] noticed that patients with an increased oxygen extraction fraction and normal or low CBV have less severe haemodynamic impairment than those with an increased oxygen extraction fraction and higher CBV. Wintermark et al. [26] described a group of severe head trauma patients with low CBF and CBV values showing preserved autoregulation or so-called pseudo-autoregulation [27]. Most likely, all patients in studied population had well-developed collateral flow as the duration of carotid artery stenosis was longer than 5 years [28], [29], [30], [31]. Due to aggressive stroke prevention with CEA and CAS, patients with chronic severe carotid stenosis are difficult to find, particularly in Western countries. Nevertheless such a population exists and might encompass a substantial number of patients in poorer countries with limited or more difficult access to expensive medical procedures. Further studies on low CBF, low CBV patients are warranted.

cc-TQ reflects changes in pial artery compliance. Increases in cc-TQ have been observed during acetazolamide and hypercapnic tests, acute hypoxia, papaverine and glucagon administration, electroconvulsive therapy and acute jugular vein insufficiency [7], [8], [9], [16], [29], while decreases in cc-TQ have been recorded during handgrip tests and following the administration of adrenaline [10], [11]. In a healthy population, there are no significant differences between hemispheres (i.e. the cc-TQ ratio between the left and right hemispheres was very close to 1.0) [32]. We hypothesized that cc-TQ may serve as a discriminating factor. Statistical analysis revealed that a cc-TQ ratio equal to 1.0 following the acetazolamide test best differentiates groups of patients with respect to CBF and CBV. Therefore we divided patients into two subgroups: the first subgroup with a cc-TQ ipsilateral/contralateral ratio ≥1 and the second subgroup with a cc-TQ ipsilateral/contralateral ratio <1. We realize that the described division of patients is somewhat arbitrary. A much larger study is required to establish the appropriate cut-off with respect to the diagnostic and prognostic use of the cc-TQ ratio.

Nevertheless, the cc-TQ ipsilateral/contralateral ratio proved to be effective in separating patients with different brain haemodynamics (Table 3, 4, 5). At baseline in the first group (cc-TQ ratio ≥1) TTP and PSV were lower on the contralateral side. PSV values are highly dependent on the degree of stenosis [33], [34]. Every attempt was made to measure PSV in the post-stenotic portion of the ICA. However, this was often difficult in patients with high division of the common carotid artery and in patients with degenerative changes in the cervical vertebrae (which made backward neck flexion impossible). Therefore it is not surprising that PSV was higher on the ipsilateral side. A lower TTP indicates that the haemodynamics was less impaired on the contralateral side. This finding is consistent with previous evidence that TTP is the most sensitive pCT parameter in detecting differences in brain haemodynamics [25], [35]. Importantly, in the first group differences with regards to cc-TQ between both hemispheres became statistically significant only after acetazolamide challenge. Patients from the first group showed a moderate increase in CBF and CBV combined with a substantial decrease in TTP and sas-TQ. On the contrary, patients from the second group showed significant differences in cc-TQ between hemispheres at baseline, but not following the acetazolamide test. CBF and CBV substantially increased after the acetazolamide challenge, while the changes in TTP and sas-TQ were not significant. We speculate that groups of patients with such differences in response to the acetazolamide test most likely differ with respect to treatment and prognosis. This however needs to be confirmed in large clinical trials.

sas-TQ reflects changes in the subarachnoid width [13]. The subarachnoid width is affected by changes in intracranial pressure, or CBV [11], [12], [16]. Interestingly, changes in sas-TQ evoked by acetazolamide were consistent with changes in TTP in both groups of patients (Table 3 and 4). TTP and sas-TQ are indirectly dependent on CBV. Nevertheless, CBV increased in all patients while sas-TQ and TTP responses differed between groups. Therefore other factors may influence these variables. Although we cannot exclude type I error due to the small sample size it is likely that TTP was affected by changes in intracranial pressure [37]. In the first group a moderate increase in CBF and CBV combined with a decrease in sas-TQ and TTP suggests elevation of intracranial pressure, which represents a typical response to acetazolamide challenge [9]. In the second group the substantial increase in cc-TQ, CBF and CBV combined with insignificant changes in sas-TQ and TTP may reflect hyperkinetic circulation without significant change in intracranial pressure [32]. Intracranial pressure might already have been elevated in this group before the acetazolamide challenge due to adaptation to high blood pressure. Hypertension and increased sympathetic drive may be associated with increased intracranial pressure (for review please see [36]).

The following limitations should be taken into account. The high within- and between-subject reproducibility and repeatability of NIR-T/BSS measurements have been demonstrated previously [10], [12], [13], [16], [32]. NIR-T/BSS, like NIRS, allows for direct within-subject comparisons [16], [35]. However, to date, measurements using IR light (NIRS and NIR-T/BSS) do not allow for direct between-subject comparisons due to differences in skull bone parameters [16], [38]. Amplitude and frequency changes were analyzed using periodogram frequency analysis. Periodograms allow for reading the peaks of the harmonic component only while peak to peak or root mean square (RMS) values are used for time waveform analysis. As long as the signal shape remains the same, amplitude changes assessed in the time domain and frequency domain are equivalent [39], [40]. We did not measure peripheral blood pressure in this study. The Diamox test typically does not evoke changes in blood pressure in humans [41] or in animals [8]. Therefore it is unlikely that such changes occurred in the present study. The absence of significant changes in HR throughout the entire test and during the presented time points also confirms that blood pressure most likely remained stable. We used high quality pCT to assess CBF and CBV instead of single-photon emission computed tomography (SPECT) or positron emission tomography (PET). pCT is a standard of care at our university hospital. Hospital radiologists are the most experienced in this imaging technic. Furthermore, pCT is one of the methods of choice for stroke assessment [42], [43]. Finally, although the pCT ROI was not located at the infarct area we cannot exclude that the cc-TQ and sas-TQ values might have been influenced by such an area.

In summary, to the best of our knowledge, this is the first publication describing changes in pial artery pulsation and subarachnoid width following an acetazolamide test in patients with chronic carotid artery stenosis. We demonstrated that the ipsilateral/contralateral cc-TQ ratio following acetazolamide challenge may be used to distinguish patient groups characterized by different haemodynamic parameters. Changes in sas-TQ following the acetazolamide test were consistent with changes in TTP. Finally we described changes in brain haemodynamics evoked by the acetazolamide test in a patient population with low CBF and CBV values. Further studies are warranted with respect to both the use of NIR-T/BSS technology in patients with cerebrovascular disorders and the patient population suffering from chronic carotid artery stenosis characterized by low CBF and CBV values.
